# Editorial: Autism Spectrum Disorders: Developmental Trajectories, Neurobiological Basis, Treatment Update

**DOI:** 10.3389/fpsyt.2017.00125

**Published:** 2017-07-13

**Authors:** Roberto Canitano, Yuri Bozzi

**Affiliations:** ^1^University Hospital of Siena, Siena, Italy; ^2^University of Trento, Trento, Italy

**Keywords:** autism spectrum disorders, neurobiology and genetics, experimental treatments, clinical and preclinical characteristics, childhood and adolescence

**Editorial on the Research Topic**

**Autism Spectrum Disorders: Developmental Trajectories, Neurobiological Basis, Treatment Update**

This Research Topic stems from the urgent need to fill the gap of the many unresolved scientific issues on autism spectrum disorders (ASD) that are still in need of investigation, and follows another Research Topic we recently edited. As an example, targeted treatments based on the understanding of the underlying pathogenic mechanisms of disease are still lacking. Further research is awaited and should be obtained through a significant effort on experimental treatment trials based on consistent “proof-of-concept” preliminary results. In the first contribution listed in this editorial, a fundamental question is raised about the current limitations in preclinical models and the need for a thorough reappraisal of their application in clinical studies of ASD. This is one of the main reflections on current investigation that we like to draw attention upon. Though we are confident that we are providing valuable contributions on different themes, we are aware that many other important issues have not been dealt with. This Research Topic has therefore major limitations and does not include all the issues that deserve further research in the vast field of ASD research. The following is an overview of the articles collected in this Topic.

Loth et al. provided an overview of current issues in ASD diagnosis and treatment. The phenotypic and etiological variability between individuals with ASD, together with the lack of effective treatments, urgently point to a precision medicine approach. Such an approach aims to identify targeted treatments based on the understanding of the underlying pathogenic mechanisms of disease that might be tackled by specific interventions, both as pharmacotherapy or behavioral intervention. To this purpose, stratification biomarkers are useful tools to select or exclude patients for a particular treatment. Further, different factors that may impact on developmental outcome have been detailed. Common variants and genetic background are to be thoroughly considered. The environmental risk factors for ASD include maternal infections and prenatal exposure to teratogenic agents such as valproate acid, exposure to toxins, and dysfunction of the immune system. Finally, the timing of impact of genetic and environmental agents on neuronal development likely plays a crucial role in determining the outcome. An important remark on factors implicated in failed clinical trials in ASD points to the limited translatability from animal models to humans. As to the new perspectives of investigation, patient-derived induced pluripotent stem cells are an interesting new tool that overcomes inter-species differences and deserves further development.

Following this line of thought, other studies investigated key gene pathways involved in ASD pathogenesis. Among the potential candidate genes identified in ASD, those involved in Akt/mammalian target of rapamycin (mTOR) signaling and the downstream effects of this pathway are highly represented including *FMR1, PTEN, TSC1*, and *TSC2*. Aberrant Akt/mTOR signaling has the potential to impact cellular growth, proliferation, and cytokine production in the immune system, which can in turn affect behavior. The activity of the mTOR pathway in cells obtained from children with ASD and typically developing controls was investigated by Onore et al. Elevated higher activity of mTOR and lower activity of glycogen synthase kinase 3 α and tuberin (TSC2) in cells from children with ASD were observed. In addition, the study showed a phosphorylation pattern supporting higher activity in the Akt/mTOR pathway in children with ASD, and not limited to known ASD-associated Akt/mTOR genetic mutations mentioned above. This common pathological pathway needs further investigation as the abnormalities in Akt/mTOR signaling observed in this study are likely not limited to T cells but would be detected also in other immune cells and have relevance to overall immune abnormalities observed in ASD. Transcriptome analyses highlighted that gene networks involved in synapse development, neuronal activity, and immune function are deregulated in ASD and prompts to carry out further research to shed light on these issues. Mouse models provide unique tools to investigate the neurobiological basis of ASD. In their study, Provenzano et al. used two well-recognized ASD mouse models, BTBR and Engrailed-2 knockout, to identify conserved clusters of ASD-related genes. Each of these clusters (modules) showed a specific enrichment profile in neuronal and glial genes, as well as in genes associated to ASD comorbidities such as epilepsy and schizophrenia. Significant transcriptional similarities and differences between the BTBR and *En2^−/−^* hippocampus were detected and thoroughly detailed. This study also underscored that transcriptome analysis of ASD mouse models may contribute to identify novel molecular targets for pharmacological studies. Indeed, ASD and schizophrenia spectrum disorders (SSD) share clinical and genetic components and co-occur more frequently than would be predicted by their respective prevalence. As detailed in the contribution of Canitano et al., a complex, multifactor association is implicated in both conditions. As a current hypothesis, social and cognitive disturbances in ASD and SSD derive from abnormalities in the ratio of excitatory to inhibitory cortical activity (E/I imbalance). Altered functions of genes coding for glutamatergic and GABAergic brain receptors and/or synaptic proteins would be at the origin of systems derangement. Current understanding of shared and divergent patterns between ASD and SSD from molecular to clinical aspects is far from clear. The Research Domain Criteria approach is promising to guide future progress and accomplishments in this field because it represents a new framework for carrying on research in neurodevelopmental disorders that diverge significantly from current standards. The aim is to build a research literature that reflects advances in genetics and neurosciences including behavioral science to provide a consistent foundation for precision diagnosis and treatment of ASD.

An original conceptualization of the emergence of language disturbances in ASD has been proposed by Benitez-Burraco et al. It has been hypothesized that language appearance would be linked to changes in the human brain/skull associated to the process of self-domestication of the human species. Individuals with ASD would exhibit less marked domesticated traits at the morphological, physiological, and behavioral levels. In addition, many ASD candidate genes are represented among the genes known to be involved in the “domestication syndrome,” e.g., the constellation of traits exhibited by domesticated mammals deriving from the hypofunction of the neural crest and also among the set of genes involved in language function. Some candidate genes for domestication and language development showed the same expression profile in people with ASD and chimps in brain areas involved in language processing. As a result, ASD may represent an abnormal ontogenetic trajectory for the human faculty of language resulting from mutations in genes important for the “domestication syndrome” and from the normal functioning of the neural crest.

Identification of molecular and structural biomarkers is crucial to identify novel, early diagnostic tools. A study on biomarkers in 94 children with ASD was carried out by Frye et al., providing important clues in relation to distinct profiles as to folate receptor autoantibodies receptors FRAA with implication for diagnosis and treatment. Folate receptor α (FRα) autoantibodies (FRAAs) are rather frequent in ASD and disrupt the transport of folate across the blood–brain barrier by binding FRα. Children positive for the binding FRAA were found to have higher serum B12 levels as compared to those negative for binding FRAAs. Conversely, children positive for the blocking FRAA were found to have relatively better redox metabolism and inflammation markers as compared to those negative for blocking FRAAs. In addition, ASD children positive for the blocking FRAA showed better communication on the Vineland Adaptive Behavior Scale, stereotyped behavior on the Aberrant Behavioral Checklist and mannerisms on the Social Responsiveness Scale. Altered zinc (Zn) homeostasis has been reported in fibroblasts from >60 years old Fragile X premutation carriers. Napoli et al. tested FMRP protein expression, brain bioenergetics, and expression of the Zn-dependent synaptic scaffolding protein SH3 and multiple ankyrin repeat domains 3 (Shank3) in a knockin premutation mouse model of Fragile X syndrome. Significant deficits in brain bioenergetics, Zn levels, and Shank3 protein expression were observed in these mice, and the authors provided evidence that in premutation carriers, altered Zn homeostasis, brain bioenergetics, and Shank3 levels could be compounded by Zn-deficient milk, increasing the risk of developing emotional and neurological/cognitive problems. Liska and Gozzi describe the progress in mouse brain connectivity mapping by means of resting-state functional magnetic resonance imaging, which opens a new avenue of investigation in ASD. This technique allows to test mechanistic hypotheses about the abnormal connections observed in ASD and examples are illustrated of how this approach can be used to establish causal links between ASD-related mutations, developmental processes, and brain connectivity architecture. The role of insulin-like growth factor 1 (IGF-1) in treating neurodevelopmental disorders including ASD is thoroughly dealt with by Vahdatpour et al. The promising potential of this polypeptide is ranging from Rett syndrome to X-fragile and ASD, due to the effects that IGF-1 exerts in the development, growth and maturation of the CNS and its synapses. Putative mechanisms of action of IGF-1 are delineated in the different disorders. In a double blind, placebo controlled Phase 2 trial, the safety and preliminary efficacy of IGF-1 treatment were reported on nine patients with Phelan and McDermid syndrome aged 5–15. The exact role of the IGF-1 in overall ASD is still unclear, as results of previous studies are conflicting. At present, clinical trials of IGF-1 in ASD are ongoing and will possibly shed light on this issue. Finally, Billeci et al. reviewed recent research findings on the neurostructural and neurofunctional substrates in parents of individuals with ASD (pASD). The primary hypothesis was that, like for the behavioral profile, the pASD express an intermediate neurobiological pattern between ASD individuals and healthy controls. The 13 reviewed studies showed that pASD are generally different from healthy controls at a structural and functional level despite often not behaviorally impaired.

Another series of studies described the importance of detailed behavioral characterization of ASD patients and the development of novel strategies of intervention. Orienting and social interest was investigated in a group of children with ASD (age >3 years) in an interesting study by Franchini et al. By means of an eye-tracking task, visual preference for social stimuli was measured in children with ASD compared to typically developing (TD) children. Reduced interest for biological motion in children with ASD compared to TD children was detected and was associated with better adaptive functioning in preschoolers with ASD. Moreover, longitudinal results showed that a preference for biological motion clearly predicted decreased severity of ASD symptoms. Eye-tracking technique confirms to be a very useful tool for collecting valuable information of developmental profile of children with ASD at early age. A thorough analysis of stereotypies in children with various disorders other than ASD was presented by Cardona et al. The complexity of stereotypies presentation urges a comprehensive standardized evaluation including developmental and clinical components that eventually provide the correct definition. Further, this approach would be fruitful to improve our knowledge of such a heterogenous field. A novel model of intervention called “Developmental and Sequenced One-to-One Educational Intervention” (DS1-EI) in 5- to 9-year-old children with ASD and intellectual disabilities (ID) was set up and presented by Tanet et al. Aim of this study was to describe a school-based intervention that was adapted to the French health and education system with the basis to implement the method and adapt it to a low-functioning population of ASD children. The treatment protocol was adapted for school implementation using an educational agenda, and the intervention was based on various principles such as intensity, regular assessments, updating objectives, encouraging spontaneous communication, promoting skills through play with peers, etc. The model was applied in 11 French institutions in small classrooms and holds promise for further application at school in this subgroup of children with ASD and ID. Bonn et al. described an automated gaming platform enabling intensive intervention in nomadic settings. The games involved application of visual and audio stimuli with multiple difficulty levels and a wide variety of tasks and actions pertaining imitation and joint attention. Performance of the platform was assessed in a 3-month open trial with 10 children with ASD, and parents highlighted enhancement in the child’s concentration, flexibility, and self-esteem in 78, 89, and 44% of the cases, respectively. This pilot study shows the feasibility of using the developed gaming platform for home-based intensive intervention.

## Author Contributions

Equal contribution by RC and YB.

## Conflict of Interest Statement

The authors declare that the research was conducted in the absence of any commercial or financial relationships that could be construed as a potential conflict of interest. The reviewer, NR, and handling Editor declared their shared affiliation, and the handling Editor states that the process nevertheless met the standards of a fair and objective review.

